# Non-coding RNAs in ossification of the posterior longitudinal ligament

**DOI:** 10.3389/fgene.2022.1069575

**Published:** 2022-11-24

**Authors:** Haoran Zhang, Qingyu Zhang, Zenong Yuan, Jun Dong

**Affiliations:** Department of Orthopedics, Shandong Provincial Hospital Affiliated to Shandong First Medical University, Jinan, Shandong, China

**Keywords:** lncRNA, circRNA, ossification of the posterior ligament, ncRNA, microRNA

## Abstract

Ossification of the posterior longitudinal ligament (OPLL) is a kind of disease that involves a variety of factors leading to ectopic bone deposition of the spinal ligament. Although the detailed mechanism is not clear, genetic factors play important roles in the development of this disease. Noncoding RNA (ncRNA) refers to an RNA molecule that is not translated into a protein but participates in the regulation of gene expression. Functionally important types of ncRNA associated with OPLL include long noncoding RNA, microRNA, and circular RNA. We listed the differentially expressed ncRNAs in OPLL patients and normal controls to find the ncRNAs most relevant to the pathogenesis of the disease. The potential regulatory networks of ncRNA in OPLL cells were analyzed based on their most abundant signal transduction pathway data. The analysis of the highly connected ncRNAs in the regulatory network suggests that they play an important role in OPLL. These findings provide new directions for the study of OPLL pathogenesis and therapeutic targets. In this paper, we reviewed and analyzed the literature on ncRNAs in OPLL published in recent years, aiming to help doctors better understand and treat this disease.

## Introduction

Ossification of the posterior longitudinal ligament (OPLL) is a disease in which fibroblasts appear in the ligament tissue and form osteoblasts due to a variety of factors. OPLL is more common in East Asia than in North America. OPLL often occurs in the cervical spine, most commonly at the C5 level (Kawaguchi et al., 2016). Cervical OPLL is also related to sex and age. The risk of cervical OPLL is higher in males than in females and higher in the elderly than in adolescents. In a report from Taiwan, the estimated prevalence of cervical OPLL was 7.7 per 100,000 person-years (Wu et al., 2011; Wu et al., 2018). CT images of 1,500 patients in Japan showed that men were 4.9% more likely than women to develop OPLL in the cervical spine, while men were 0.6% less likely than women to develop OPLL in the thoracic spine (Fujimori et al., 2016). Although the underlying mechanism of OPLL has not been thoroughly studied, recent theories suggest that OPLL is associated with genetic factors (Nishida et al., 2015).

Noncoding RNA (ncRNA) refers to a class of RNA transcribed from the genome that does not encode proteins, and there are tens of times more of them than protein-coding RNAs. In the past decade, ncRNAs have been regarded as useless “junk” RNAs, but in recent years, research has shown that ncRNAs play a crucial role in regulating gene expression as information carriers, potential biomarkers, and therapeutic targets (Han et al., 2013; Kopp and Mendell, 2018; Tsuru et al., 2018). There are many kinds of functionally important ncRNAs that form ncRNA action networks through the permutations and combinations of secondary and/or tertiary structures (non-coding RNA interactor networks/NINs) and play important roles in the development of OPLL (Fabbri et al., 2019).

At present, some reports have found that ncRNAs regulate ossification and further influence OPLL progression. For example, miR-146a may inhibit osteogenic differentiation by blocking tumor necrosis factor (TNF)-α activation of nuclear factor κB (NF-κB) in bone mesenchymal stem cells (BMSCs) derived from human adipose tissue (Cho et al., 2010). TNF-α-induced NF-KB activation stimulates the growth of microRNA-150-3p and inhibits the osteogenic differentiation of mesenchymal stem cells by targeting β-catenin (Wang et al., 2016). Currently, we have very little knowledge of ncRNAs in patients with OPLL, both in terms of quantity and mechanism of action. The purpose of our study was to identify some ncRNAs and mRNAs that are most correlated with OPLL, to study their roles in the progression of OPLL according to their targets and to obtain the potential therapeutic value of these ncRNAs and mRNAs for OPLL.

## LncRNAS involved in ossification of the posterior longitudinal ligament

### Aberrantly expressed lncRNAs of osteogenically differentiated mesenchymal stem cells in ossification of the posterior longitudinal ligament

Cai et al. (2020) investigated the interaction between abnormally expressed lncRNAs and mRNAs in OPLL mesenchymal stem cells and predicted lncRNA targets and transcription factors. They found 651 upregulated lncRNAs and 74 downregulated lncRNAs in the MSCs of the OPLL patients (Table 1). They also used the top 10 differentially expressed lncRNAs and mRNAs for reverse transcription quantitative polymerase chain reaction (RT-qPCR) assays, and the results confirmed the microarray data. Interestingly, the authors found that in patients with OPLL, the most important upstream potential transcription factor of lncRNA was Octamer transcription factor 1 (Oct-1), which had the most interactions. Oct1 is widely distributed in tissues. It is a complex and versatile transcription factor that plays an essential role in maintaining cellular homeostasis and signaling pathways. It regulates the underlying expression of genes by recruiting and activating RNA polymerase III. Moreover, Oct1 is directly linked to the core transcription mechanism. However, it can also induce pauses to balance genes for rapid transcription (Pance, 2016). Oct1 may be an important target for studying the regulation of OPLL transcription in the future, and many mechanisms have not been thoroughly studied.

**TABLE 1:** LncRNAs expression profiles in OPLL.

No.	Evaluation method	Sample	Dysregulation (up)	Dysregulation (down)	Signal pathway	References
1	Microarray	Human MSCs	651 lncRNAs	74 lncRNAs	—	Cai et al. (2020)
	RT-qPCR			LncRNA-p26842		
2	RNA-sequencing	PBMCs	751 lncRNAs	650 lncRNAs	—	Jiang et al. (2021)
3	qRT-PCR	Ligament fibroblasts	LncRNA XIST	—	Targets miR-17-5p to promote the expression of AHNAK and BMP2	Liao et al. (2019)
4	lncRNA microarray	Ligament fibroblasts	73 lncRNAs	69 lncRNAs	—	Wang et al. (2020)

Not only upstream transcription factors but also some LncRNAs can affect the relationship between the bone morphogenetic protein-9-guided signal transduction pathway and the receptor and ligand of Notch, thus affecting the osteogenic differentiation process of MSCs. Zhang et al. (2019) found that lncRmst knockout reduces the expression of Notch receptors and ligands to inhibit Bmp9-induced osteogenic, chondrogenic and adipogenic differentiation and weakens BMP9-induced ALP activity and ectopic formation of bone matrix. BMP9 can directly induce osteogenic differentiation through Notch or other mediators. However, the ligand and receptor of Notch are inhibited by some microRNAs, such as miR-107, miR-125a, miR-27b, miR-34a, miR-449a and miR-449b. LncRmst can sponge microRNAs to promote osteogenic differentiation, such as miR-106, miR-125a, miR-449a and miR-449b. Not coincidentally, other researchers have found that lncRNA H19 and HOTAIR are closely associated with BMP9-induced osteogenic differentiation in MSCs. LncRNA H19 affects the early stages of BMP9-induced osteogenic differentiation by regulating Notch ligand expression, and either silencing or overexpression of lncRNA H19 inhibits BMP9-triggered terminal osteogenic differentiation of MSCs. However, activation of Notch signaling in turn rescues the inhibited osteogenic differentiation (Liao et al., 2017).

### LncRNA expression profiles in peripheral blood mononuclear cells

Jiang et al. (2021) profiled the differential lncRNA expression from peripheral blood mononuclear cells (PBMSCs) and identified 751 upregulated and 650 downregulated lncRNAs by using RNA-sequencing. The lncRNA with the most upregulated expression was LNC-Cryba4-1:21, which was upregulated 1790-fold. The lncRNA with the most downregulated expression was lncILF3-AS1, which was downregulated 1185-fold. To prove the authenticity of the RNA sequencing results, the authors randomly chose two upregulated lncRNA groups and two downregulated lncRNA groups to perform RT-qPCR. The results of RT-qPCR reconfirmed the results of RNA sequencing. LncRNA ILF3-AS1 (ILF3-AS1) has been reported to be abnormally expressed in some tumors. Hu et al. (2019) found that lncIlF3-AS1 levels were significantly upregulated by the nuclear transcription factor SP1 in osteosarcoma tissues and cells. Moreover, ILF3-AS1 plays a tumor-promoting role in the progression of osteosarcoma through the miR-212/SOX5 axis. LncILF3-AS1 is a ceRNA (competing endogenous RNA) of miR-212, and overexpression of ILF3-AS1 can significantly reduce the expression of miR-212 and increase the expression of SOX5. However, downregulation of ILF3-AS1 inhibited the proliferation, migration and invasion of osteosarcoma cells and ultimately promoted their apoptosis. Some mechanisms of lncRNA ILF3-AS1 have been explored in bone tumors, but its specific role in OPLL is still unclear, which may be a research direction for further development in the future.

### LncRNA expression profiles in primary human ligament fibroblasts

Liao et al. (2019) found that lncRNA X inactive specific transcript (XIST) was significantly increased in primary human ligament fibroblastic cells from OPLL tissues. Some researchers have found that lncRNA XIST regulates miR-17-5p/AHNAK/BMP2 through a signaling pathway to affect posterior longitudinal ligament ossification. Significantly higher levels of lncRNA XIST in response to mechanical stress led to lower levels of miR-17-5p. This further led to increased levels of AHNAK, BMP2 and runt-related transcription factor 2 (RUNX2), which promoted osteogenic differentiation of posterior longitudinal ligament cells. METTL3 affects the osteogenic differentiation of primary ligament fibroblasts *via* the lncRNA XIST/miR-302a-3p/USP8 axis. METTL3 not only increases the expression level of the lncRNA XIST but also promotes the m6A methylation modification of lncRNA (Yuan et al., 2021). For example, Song et al. (2021) found that METTL3 can activate the mitogen-activated protein kinase (MAPK) signaling pathway by regulating the m6A modification and expression of a lncRNA. This enhances the osteogenic differentiation of human adipose-derived stem cells. However, miR-302a-3p can in turn affect METTL3 levels through a negative feedback mechanism. Wang et al. (2020) reported that 73 lncRNAs were upregulated and 69 lncRNAs were downregulated in ligament fibroblasts from OPLL tissues. In particular, the greatest upregulated small nucleolar RNA host gene 1 (SNHG1) expression was confirmed *via* RT-qPCR; even more surprising was the authors’ finding that SNHG1 acts as a sponge for miR-320b. This led to increased expression of interferon gamma receptor (IFNGR1) and activation of the JAK/STAT signaling pathway, ultimately leading to increased ligament fibroblast mineralization and osteogenic differentiation.

## MiRNA involved in ossification of the posterior longitudinal ligament

### MiRNA expression profiles in primary ligament cells

By comparing the primary posterior longitudinal ligament cells of OPLL and normal PLL, Xu et al. (2016) demonstrated that 144 miRNAs were upregulated and 74 miRNAs were downregulated (Table 2). After verification, it was found that the expression of Runx2 and IBSP in the miR-10a-5p overexpression group was the most upregulated, and had a strong role in promoting bone matrix mineralization and calcium deposition. Jiang et al. (2020) found that miR-497 and miR-195 were downregulated in PLL tissues of OPLL patients, while the expression levels of ADORA2A were significantly increased. Mechanical stress can promote the process of osteogenic differentiation. The interesting finding is that the expression of miR-195 and miR-497 is also reduced in PLL cells treated with cyclic mechanical stress (CMS). This is because CMS significantly enhances CpG island methylation of the miR-195 and miR-497 promoters in PLL cells. miR-497-5p has been reported to be a modulator of cartilage-related genes (Hou et al., 2019). Moreover, BMSCS-EVs can upregulate miR-497-5p levels to reduce RSPO2 levels and thus inhibit the Wnt/β-catenin pathway to promote osteogenic differentiation. Ma et al. (2020) found that serum levels of miR-497-5p are significantly decreased in postmenopausal women, and overexpression of miR-497-5p promotes osteogenic differentiation and bone mineralization. Liu et al. (2020) found that miR-181a-5p and miR-181a-3p were highly expressed in OPLL ligament cells, and the level of miR-181a-5p in OPLL was significantly higher than that of miR-181a-3p compared with normal PLL primary ligament cells. MiR-181a-5p and miR-10a-5p have a similar mechanism of action, both of which have a strong role in promoting osteogenic differentiation. Moreover, miR-181a-5p significantly promoted ALP activity and calcium deposition after overexpression of the microRNA mimic, but miR-181A-3p had little overexpression effect. The use of modified miRNA antisense inhibitors (antagomirs) also confirmed that miR-181a-5p plays a major role in regulating cell ossification *in vitro*. *In vivo*, the authors further demonstrated that miR-181a-5p significantly increased the osteogenic capacity of posterior ligament cells and led to increased cervical osteophyte formation in Tip-Toe-walking OPLL mice. By means of RT‒PCR, Zhang et al. (2017) found that the expression of miR-563 in OPLL cultured ligament cells was significantly higher than that in PLL ligament cells. After the miR-563 overexpression treatment, the expression levels of the osteogenic-related genes RUNX2, IBSP and ALP were higher than those in the control group. After miR-563 inhibition treatment, the levels of osteoblast-related genes decreased. Furthermore, the mechanism of action of miR-563 was further studied. The high expression of miR-563 can reduce the expression of SMURFl, thus stabilizing SMAD protein, promoting the activity of BMP2 and other pathways, and ultimately promoting osteogenic differentiation.

**TABLE 2:** MiRNAs expression profiles in OPLL.

No.	Evaluation method	Sample	Dysregulation (up)	Dysregulation (down)	Signal pathway	References
1	RNA-sequencing	primary ligament cell	144	74	—	Xu et al. (2016)
2	RT-qPCR Western blot	OPLL cells	—	miR-195	Targets ADORA2A to inhibit the cAMP/PKA pathway	Jiang et al. (2020)
3	RT-qPCRWestern blot	OPLL cells	—	miR-497	Targets ADORA2A to inhibit the cAMP/PKA pathway	Jiang et al. (2020)
4	RT-qPCRWestern blot	OPLL cells	miR-181a-5p	—	Targets PBX1 and ACAN	Liu et al. (2020)
5	RT-qPCRWestern blot	OPLL cells	miR-181a-3p	—	—	Liu et al. (2020)
6	High through-put microrna sequencing	OPLL blood samples	miR-563	—	Targets SMURF1to stabilize SMAD protein and promote the activity of BMP2	Zhang et al. (2017)
14	High through-put microrna sequencing	OPLL blood samples	miR-10a-3p	—		Xu et al. (2019)
15	High through-put microrna sequencing	OPLL blood samples	miR-10a-5p	—	—	Xu et al. (2019)
7	High through-put microrna sequencing	OPLL blood samples	miR-210-3p	—	Targets MAPK to inhibit osteogenic differentiation	Xu et al. (2019)
8	High through-put microrna sequencing	OPLL blood samples	miR-885-5p	—	Targets β-catenin	Xu et al. (2019)
9	High through-put microrna sequencing	OPLL blood samples	—	miR-129-2-5p	—	Xu et al. (2019)
10	High through-put microrna sequencing	OPLL blood samples	—	miR-199b-5p	Targets JAG1 and modulates the Notch signaling pathway to inhibits osteogenic differentiation	Xu et al. (2019)
11	High through-put microrna sequencing	OPLL blood samples	—	miR-212-3p	Targets GDF5 and further activates Smad1/5/8 phosphorylation to inhibits osteogenic differentiation	Xu et al. (2019)
12	High through-put microrna sequencing	OPLL blood samples	—	miR-196b-5p	Targets FGF2	Xu et al. (2019)
13	High through-put microrna sequencing	OPLL blood samples	—	miR-218-1-3p	—	Xu et al. (2019)
16	Edger package of R software	OPLL cell specimens	80	264	—	Xu et al. (2020)
17	High through-put microrna sequencing	OPLL cells	miR-320e	—	Targets TAK1 to promote osteogenic differentiation	Xu et al. (2022)
18	High through-put microrna sequencing	OPLL cells	miR-6720-3p	—	—	Xu et al. (2022)
19	High through-put microrna sequencing	OPLL cells	miR-3185	—	—	Xu et al. (2022)
20	RT-qPCRWestern blot	OPLL tissue	miR-497-5p	—	Targets RSPO2 to inhibit the Wnt3α/β-catenin pathway	Chen et al. (2021)
21	microRNA array	OPLL cells	—	12	—	Yayama et al. (2018)

### The miRNA expression profiles in blood

Based on the high-throughput miRNA sequencing data of OPLL and PLL cells, Xu et al. (2019) selected 10 miRNAs with the largest expression differences. Among the 10 selected miRNAs, miR-10a-3p, miR-10a-5p, miR-563, miR-210-3p and miR-885-5p were highly upregulated in OPLL tissues. However, miR-129-2-5p, miR-199b-5p, miR-196b-5p, miR-212-3p and miR-218-1-3p were highly downregulated. In addition, miR-10a-5p, miR-563 and miR-210-3p showed high accuracy and significance in patients who had OPLL in the normal group and IDD (intervertebral disc degenerated) group. However, the authors found no significant difference in serum bone metabolites in differentiating OPLL from IDD and non-IDD patients, so the determination of these miRNAs may play an important role in the early detection of OPLL. Moreover, a combination of three miRNAs is more diagnostic than a single miRNA. Significant increases in miR-10a-5p and miR-10a-3p were also found in other studies involving OPLL. This suggests that these two miRNAs are closely associated with the progression of OPLL and may have the potential to function as hub RNAs in a complex mechanism of action.

### The miRNA expression profiles in mesenchymal stem cells

Xu et al. (2020) analyzed a total of 344 differentially expressed miRNAs from the database by using the edgeR Package of R Software, among which 80 miRNAs were upregulated and 264 were downregulated. More interestingly, they identified miR-520d-3p, miR-4782-3p, miR-6766-3p and miR199b-5p as hub miRNAs by FunRich prediction. These miRNAs have been shown to be associated with the development of hepatocellular carcinoma, lung cancer and peripheral vascular disease in reported studies, but their specific mechanisms in OPLL are still poorly understood. It is only known that SP1 can act as an upstream regulator and participate in the process of posterior longitudinal ligament ossification *via* the Wnt signaling pathway through LEF1 and Wnt2.

An interesting phenomenon was found by Xu et al. (2022) Although the level of miR-320e was upregulated in OPLL cells, it was far less than that in OPLL mesenchymal stem cell-derived exosomes. This indicates that miRNAs strongly associated with certain diseases can spontaneously accumulate in sEVs and be transmitted to other cells to exert their effects. The authors also found that miR-6720-3p and miR-3185 were also significantly upregulated, similar to miR-320e. However, the exact mechanism of their action is not yet understood, and may be a direction for future research. Chen et al. (2021) reported that mesenchymal stem cells from fat and muscle tissue have high osteogenic differentiation ability. Immunofluorescence showed that mesenchymal stem cells released extracellular vesicles and were entoxified by ligament fibroblasts to upregulate miR-497-5p. Yayama et al. (2018) detected a total of 177 microRNA factors through the microRNA array, and the expression of 12 factors in OPLL cells was significantly downregulated compared with non-OPLL cells in the control group (CSM patients). Hsa-miR-487b-3p is downregulated in OPLL patient cells, and the expression of genes involved in Wnt signal transduction may be related to this RNA. Wnt signaling regulates the osteogenic differentiation of chondrocytes and osteoblasts and regulates cartilage osteogenesis in the pathogenesis of OLF and posterior longitudinal ligament ossification.

In OLF cells, Han et al. (2018) revealed that miRNA-342-3p was significantly elevated during osteogenic differentiation and significantly decreased during bone loss by miRNA sequencing analysis. MiRNA-342-3p promotes osteogenic differentiation by directly targeting ATF3 and inhibiting ATF3 inhibition. After miRNA-342-3p was knocked out in human mesenchymal stem cells (MSCs), the ALP, osteocalcin (OCN), COL I and RUNX2 genes related to osteogenesis were downregulated. Although there are no research results of miR-342-3p in OPLL, its role in promoting osteogenic differentiation in OLF and MSCs is sufficient for us to conduct correlation studies. In addition, miR-342-3p may be a vital potential target for the treatment of OPLL in the future.

## CircRNA involved in ossification of the posterior longitudinal ligament

### CircRNA expression profiles in peripheral blood mononuclear cells

Jiang et al. (2021b) reported that 177 circRNAs were upregulated and 154 circRNAs were downregulated in peripheral blood mononuclear cells (PBMCs) (Table 3). The circRNA with the most upregulated expression was hsa-circ-0092429, which was upregulated 38.78-fold. The circRNA with the most downregulated amount was hsa-circ-0137606, which was downregulated 292-fold. To prove the authenticity of the RNA sequencing results, the authors randomly choose two upregulated circRNA groups and two downregulated circRNA groups to perform RT-qPCR. The results of RT-qPCR reconfirmed the results of RNA sequencing.

**TABLE 3:** CircRNAs expression profiles in OPLL.

No.	Evaluation method	Sample	Dysregulation (up)	Dysregulation (down)	Signal pathway	References
1	RNA-sequencing RT-qPCR	PBMCs	177 circRNAs	154 circRNAs	—	Jiang et al. (2021a)
2	OPLL tissue	72 circRNAs		74circRNAs	—	Han et al. (2018)
3	qRT-PCR	OPLL tissue	circSKIL	—	Promotes the expression levels of RUNX2, ALP, COL I, OCN and OPN *via* JNK/STAT3 pathway	Jiang et al. (2021b)

### CircRNA expression profiles in ossification of the posterior longitudinal ligament cells

By comparing OPLL tissue samples and non-OPLL tissue samples, Jiang et al. (2021a) demonstrated that 72 circRNAs were upregulated and 74 circRNAs were downregulated. Moreover, their follow-up study showed that hsa-circ-0007292 was the most elevated in OPLL cells. MiR-508-3p can inhibit the expression of osteogenesis-related genes by inhibiting SATB2 mRNA expression. Hsa-circ-0007292 is most commonly found in the cytoplasm and acts as a sponge for miR-508-3p to promote the progression of OPLL.

### CircRNA expression profiles in primary posterior longitudinal ligament cells

Sun et al. (2021) found that circSKIL expression levels were significantly elevated in primary cervical posterior longitudinal ligament cells from patients with OPLL and promoted osteogenic differentiation through the JNK/STAT3 pathway. In addition, the overexpression of circSIKL can also improve the expression levels of RUNX2, ALP, COL I, OCN and OPN, thus proving the role of circSIKL in the ossification of the posterior longitudinal ligament.

## The classic pathways in patients with ossification of the posterior longitudinal ligament

### The classical pathways of bone mesenchymal stem cells

**FIGURE 1:**
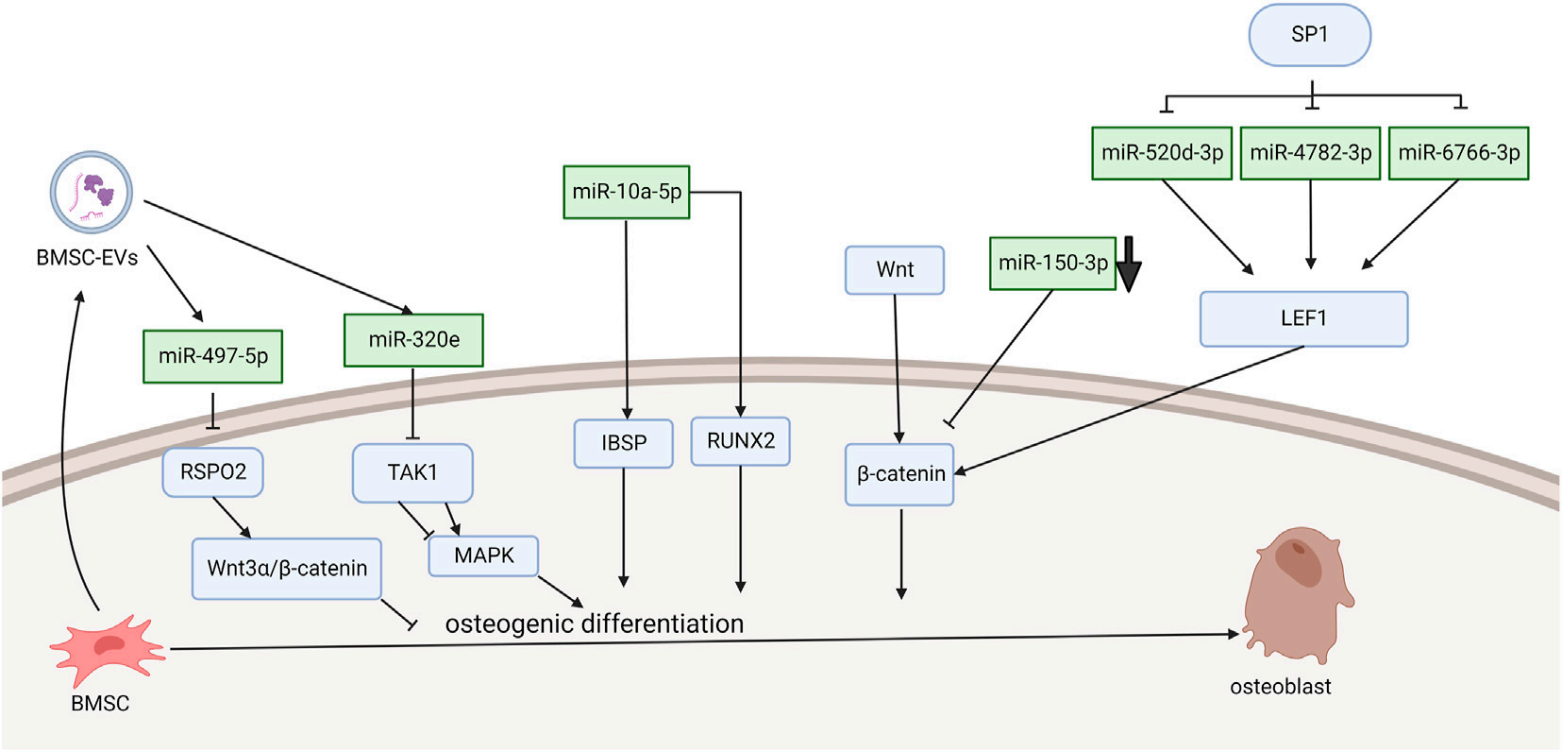
Functions of specific ncRNAs in bone marrow mesenchymal stem cells. (“Created with BioRender.com”).

Xu et al. (2016) demonstrated that miRNA-10a-5p expression activated Runx2 and Ibsp signaling, which promoted osteogenic differentiation (Figure 1). They found that PLL cells can transdifferentiate into an osteoline by Alizarin Red staining. The SP1 transcription factor leads to OPLL through the WNT signaling pathway, and this process is achieved by downregulating miR-520d-3p, miR-4782-3p, and miR-6766-3p to stimulate LEF1 overexpression (Xu et al., 2020). Wang et al. (2016) demonstrated that TNF-α can upregulate the level of miR-150-3p. Moreover, there is a complementary sequence region on the 3′-UTR of β-catenin, which is predicted to bind specifically to miR-150-3p and reduce the level of β-catenin. The following literature provides the new research idea that microRNA delivery by extracellular vesicles plays a crucial role in the formation of OPLL. Chen et al. (2021) found that immunofluorescence showed that mesenchymal stem cells released extracellular vesicles and were entoxified by ligament fibroblasts. Bone marrow mesenchymal stem cell-derived extracellular vesicles (BMSC-EVs) upregulate miR-497-5p and target the reverse regulation of RSPO2. However, RSPO2 can activate the Wnt3α/β-catenin pathway to inhibit osteogenic differentiation. Therefore, BMSCS-EVS promotes osteogenic differentiation through upregulation of miR-497-5p and then targets downregulation of RSPO2 to reduce the Wnt3α/β-catenin pathway. Moreover, Western blot results showed that the expression of osteogenic genes (BMP2, ALP, Collagen I, Osteocalcin, and Osteopontin) in BMSC-EV-treated ligament fibroblasts was upregulated. This conclusion is different from the well-known function of the WNT signaling pathway. Moreover, Xu et al. demonstrated in more detail one of the mechanisms by which exosomes promote ossification of the posterior longitudinal ligament. They discovered that miR-320e was more abundant in both OPLL cells and exosomes than in PLL. In their study, miR-320e targeted TAK1 and significantly downregulated it, thereby promoting osteogenic differentiation and inhibiting osteoclast formation. Different studies suggest that TAK1 can both promote and inhibit osteogenic differentiation. Therefore, TAK1 is considered to be a bidirectional regulator of early and late osteogenic differentiation.

### Classical pathways and functions of mechanical stress in primary ligament fibroblasts

**FIGURE 2:**
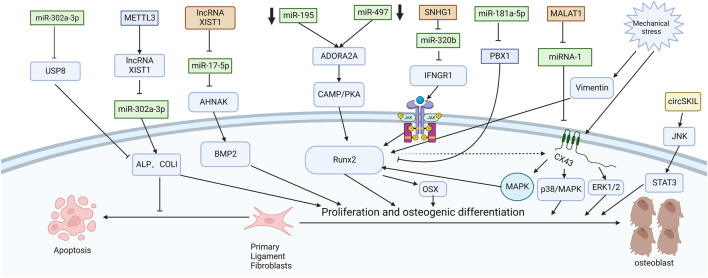
Functions of specific ncRNAs in ligament fibroblasts. (“Created with BioRender.com”).

Chen et al. (2016) explored the mechanism of promoting osteogenic differentiation from the perspective of mechanical stress. They established an *in vitro* stress loading model. Connexin 43 (Cx43) was highly expressed in the posterior longitudinal ligament fibroblasts of OPLL patients. The extracellular regulated protein kinase (ERK) 1/2 protein, p38 MAPK protein and JNK protein were significantly increased in both OPLL and non-OPLL cells after mechanical loading. Compared with siRNA interference of Cx43, blocking the ERK1/2 or p38 MAPK pathway alone only partially weakened the osteogenic effect of mechanical stress, while blocking the JNK pathway alone had no significant difference. These results suggest that the ERK1/2 and p38 MAPK pathways mediate Cx43 osteogenesis in ligamentous fibroblasts to some extent, while the JNK pathway has little correlation with Cx43 osteogenesis (Figure 2). Moreover, Yang et al. (2016) found that downregulation of connexin 43 could inhibit the osteogenic differentiation induced by dexamethasone. This is achieved by inhibiting the activity of RUNX2 and ERK and decreasing the expression of bone genes. Zhang et al. (2014) examined the effects of mechanical stress on vimentin and osteogenic genes by Western blotting and semiquantitative reverse transcription-polymerase chain reaction. They found that mechanical stress-induced vimentin downregulation in ligament fibroblasts further increased OCN, ALP, and COL I expression. Ultimately, it promoted fibroblast ossification, leading to OPLL.

CircSKIL promoted the proliferation and differentiation of primary posterior longitudinal ligament fibroblasts through the JNK/STAT3 pathway. Liao et al. showed that lncXIST was significantly increased in primary human ligament fibroblastic cells from OPLL tissues. This reduced the expression level of miR-17-5p and increased the levels of AHNAK and BMP2, which promoted the proliferation and differentiation of primary posterior longitudinal ligament fibroblasts.

Wang et al. reported that the lncRNA SNHG1 could inhibit the expression of miR-320b to upregulate IFNGR1 by serving as a sponge for miR-320b. Moreover, it activates the JAK/STAT pathway. When the authors silenced SNHG1, the osteogenic differentiation and mineralization of ligament fibroblasts were reduced.

METTL3 inhibited the expression of miR-302a-3p by upregulating lncRNA XIST and ultimately induced the expression of ALP and COL I to promote osteogenic differentiation of primary ligament fibroblasts (Yuan et al., 2021). MALAT1 promoted osteogenic differentiation of primary ligament fibroblasts by inhibiting the inhibitory effect of miR-1 on Connexin-43 (Yuan et al., 2019). After PLL fibroblasts were successfully isolated, Jiang et al. (2020) used siRNA to specifically silence ADORA2A and administered CMS treatment. The expression of osteogenic factors (ALP, OCN, Runx2, COL I) and calcium content were significantly decreased in the si-Adora2A group. Moreover, the cAMP/PKA pathway inhibitor H89 confirmed that ADORA2A could activate the cAMP/PKA pathway. Therefore, the downregulation of miR-497 and miR-195 leads to the elevation of ADORA2A, which then activates the cAMP/PKA pathway to increase the expression of osteogenic factors and promote the formation of OPLL. Liu et al. (2020) first demonstrated that miR-181a-5p, rather than miR-181a-3p, directly targeted PBX1 and ACAN in ligament cells and reduced their levels by a luciferase reporter assay. After miR-181a-5p was inhibited, ALP activity was restored only after PBX1 was knocked out. PBX1 overexpression also downregulated the level of miR-181a-5p. After ACAN was knocked out, the activity of ALP could not be restored after miR-181a-5p was inhibited. These results further demonstrate the indispensable role of PBX1 in the OPLL process and the unique relationship between miR-181a-5p and PBX1. In patients with ankylosing spondylitis, the Wnt/β-catenin pathway is also found to play an important role. Tang et al. (2018) found that miR-124 increased and GSK-3βdecreased in AS ligament tissue. Upregulation of miR-124 significantly reduced the expression of GSK-3β in osteoblast differentiation, weakened the inhibition of Wnt/β-catenin pathway activity, enhanced the expression of Osterix and RUNX2, and promoted osteoblast differentiation of ligament fibroblasts. Some studies have also shown that the Wnt/β-catenin signaling pathway can immediately promote osteoblast differentiation by upregulating the expression of osteoblast-specific genes such as RUNX2 (Kugimiya et al., 2007), Osterix and ALP and downregulating the expression of CCAAT/enhancer-binding protein alpha (CEBPα) and peroxisome proliferator-activated receptor gamma (PPARγ) (Qiu et al., 2007; Cawthorn et al., 2012). It inhibited the differentiation of mesenchymal fibroblasts into adipocytes and indirectly promoted the differentiation of mesenchymal fibroblasts into osteoblasts.

## Challenges and perspectives

OPLL is a common spinal surgical condition in which the posterior longitudinal ligament at the posterior border of the vertebral body ossifies and causes a series of clinical symptoms due to compression of the spinal cord or nerve roots under the influence of various related factors. The patient’s condition will worsen, especially when subjected to external forces, which seriously affects the patient’s daily life. However, OPLL is highly genetically heterogeneous and has a complex formation mechanism, which is still not understood. Currently, there is no effective treatment except surgery. With the advancement of sequencing technology and the rapid development of bioinformatics, the etiology of OPLL and the establishment of therapeutic targets are also flourishing.

The current understanding of the regulatory roles of different lncRNAs, miRNAs and circRNAs in the osteogenic differentiation of OPLL cells can be used to prevent or treat OPLL. For example, some circRNAs act as sponges of miRNAs to inhibit the osteogenic differentiation effect of miRNAs. Alternatively, some ncRNAs that promote osteogenic differentiation are used to treat osteoporosis. However, there is no relevant report on the clinical application of ncRNA, and the safety and efficacy of clinical research should be the focus of future studies. If scholars in the future create large-scale deep phenotypic biobanks and map the characterization of the relationship between genome and disease, this can provide a strong theoretical basis for the study of disease etiology. When the genotype of the relevant potential drug target is presented, the biological rationale of clinical research on the drug will also be determined.

## Conclusion

In general, great progress has been made in the study of the regulatory mechanism of ncRNA in the development of OPLL. The deep mining of ncRNA provides a good basis for OPLL research both in predicting the pathogenesis and in treating it. Although lncRNA and miRNA have been studied the most among ncRNAs, there are still many underlying mechanisms of osteogenic differentiation of OPLL cells that have not been clarified, especially circRNA and siRNA. In summary, ncRNAs mainly regulate the mechanism of OPLL in the following aspects. First, ncRNA can regulate gene expression at the epigenetic level. For example, lncRNAs can promote DNA methylation to promote bone formation. Second, ncRNA regulates OPLL at the transcriptional level, and miRNA-10a-5p expression activates osteogenic transcription factor Runx2 signaling, thereby promoting osteogenic differentiation. Finally, ncRNA can regulate OPLL progression through endogenous competition. For example, lncRNA, as a ceRNA of miRNA, is adsorbed on miRNA as a sponge, thus inhibiting miRNA expression.

In this paper, we reviewed the recent literature on OPLL-related RNA research at home and abroad, analyzed the impact of OPLL on its occurrence and development, and briefly introduced the shortcomings of current research and the latest research trends on OPLL susceptibility genes. We also present a preliminary investigation of the potential ossification mechanism of OPLL and discuss the effects of mechanical stress and exosomes on OPLL.

## Abbreviations

BMP-2, Bone morphogenetic protein-2; BMSC, Bone mesenchymal stem cells; BMSC-sEVs, Bone marrow mesenchymal stem cell-derived small extracellular vesicles; Col 1, Collagen 1; ERK, Extracellular regulated protein kinases; IFNGR1, Interferon gamma receptor; MAPK, Mitogen-activated protein kinase; miR, MicroRNA; OCN, Osteocalcin; OSX, Osterix; RT-qPCR, Reverse transcription quantitative polymerase chain reaction; RUNX2, Runt-related transcription factor 2; SNHG1, Small nucleolar RNA host gene 1; STAT, Signal transducer and activator of transcription.
